# Weak clinical effect of airway clearance on ADH and BNP concentration: mechanism or data collection BIAS

**DOI:** 10.1590/S1677-5538.IBJU.2023.0138

**Published:** 2023-05-20

**Authors:** Mateus Augusto Felix de Melo, Pâmela Marillac Rodrigues Feijó de Melo, Ially Raissa de Sá Gonçalves, Cecília Beatriz Gomes Marques, Beatriz Santos Moraes da Cunha Bezerra, Johnnatas Mikael Lopes

**Affiliations:** 1 Centro Universitário Uninassau Paulo Afonso BA Brasil Centro Universitário Uninassau, Paulo Afonso, BA, Brasil; 2 Universidade Federal do Vale do São Francisco Petrolina Pernambuco Brasil Universidade Federal do Vale do São Francisco - UFVSF, Petrolina, Pernambuco, Brasil

To the editor,

Nocturnal enuresis is a disorder characterized by intermittent urinary incontinence that occurs during periods of sleep for at least one episode per month for at least 3 months (1). These episodes occur at a time when the child should, under normal circumstances, be able to control urination. In addition, although some causes are more common, such as lack of pelvic floor muscle control (2) and the presence of psychiatric disorders (3), there are also pathophysiological factors that can lead to the onset of nocturnal enuresis, such as obstruction of the upper airways (4).

This pathophysiological picture is captured by sympathetic receptors of the Autonomic Nervous System, which cause greater cardiomyocyte activity, secreting B-type natriuretic peptide (BNP) by the ventricles, which stimulates natriuresis and diuresis in order to compensate for the vasoconstrictor systems. that are activated in these situations. This increase in BNP, due to the pathophysiological condition of OSA, causes inhibition of the secretion of antidiuretic hormone (ADH), which regulates diuresis through the reabsorption of water in the collecting ducts (4).

The study by Ribeiro et al. (5), published in this journal in issue 48 (6), presents interesting results on enuresis. However, we bring an analysis based on clinical effect measurement to complement the probabilistic analysis presented by the authors. The measure of the clinical effect used is the Cohen's d, whose meaning is used to find out the usefulness of the probabilistic effect, which is influenced by the sample size and selection method (6).

The improvement shown for nocturnal enuresis in the study by Ribeiro et al. (5) was clinically very large (d=1.50) compared to the effects of surgery on the concentration of ADH (d=0.38) and BNP (d=0, 32), which are considered small clinical effects, even the comparison before and after BNP being significant from the probabilistic point of view.

From this, due to the paradoxical effect of ADH and BNP, some considerations of the mechanism of effect of upper airway obstruction on nocturnal enuresis in children arise: the small clinical effect of the increase in BNP is enough to strongly increase the proportion of nights dry? Can the delay time in data collection between 90 and 120 days affect hormonal indicators mainly in the heterogeneity of the sample? Is there another factor involved?

Therefore, we can conclude that airway clearance modifications had little clinical impact on the hormonal actions of ADH and BNP as collected.

The Authors[Table t1]

**Table 1 t1:** Measures of the clinical effect of upper airway clearance on enuresis and BNP and ADH hormones.

	X_before_	X_after_	SD_before_	SD_after_	X_before_-X_after_	SD_m_	N	Square SD	Cohen's d
BNP (pg/mL)	116.5	156.2	126.5	112.3	-39.7	716291.05	49	120.90	-0.32
Dry nights %	32.3	75.4	24.7	33.4	-43.1	40003.81	49	28.57	-1.50
ADH (pg/mL)	5.8	14.6	3.2	35.4	-8.8	25360.16	49	22.74	-0.38

ADH = anti-diuretic hormone; BNP = brain natriuretic peptide; X = Mean; SD = Standard deviation; N = sample number.
